# Design paper: A phase II study of Bevacizumab and Erlotinib in patients with non-Squamous non-small cell lung cancer that is refractory or relapsed after 1-2 previous Treatment (BEST)

**DOI:** 10.1186/1745-6215-12-120

**Published:** 2011-05-12

**Authors:** Shiro Tanaka, Yuichi Sakamori, Miyuki Niimi, Megumi Hazama, Young H Kim, Kazuhiro Yanagihara

**Affiliations:** 1 Department of Clinical Trial Design and Management, Translational Research Center, Kyoto University Hospital, 54 Shogoin Kawahara-cho, Sakyo-ku, Kyoto 606-8507, Japan; 2 Department of Respiratory Medicine, Graduate School of Medicine, 54 Shogoin Kawahara-cho, Sakyo-ku, Kyoto 606-8507, Kyoto University, 606-8507, Japan; 3 Outpatient Oncology Unit, Kyoto University Hospital, 54 Shogoin Kawahara-cho, Sakyo-ku, Kyoto 606-8507, Japan; 4 Department of Translational Clinical Oncology, Graduate School of Medicine, 54 Shogoin Kawahara-cho, Sakyo-ku, Kyoto 606-8507, Kyoto University, 606-8507, Japan

## Abstract

**Background:**

Combination of erlotinib and bevacizumab is a promising regimen in advanced non-squamous non-small-cell lung cancer (NSCLC). We are conducting a single arm phase II trial which aims to evaluate the efficacy and safety of this regime as a second- or third-line chemotherapy.

**Methods:**

Key eligibility criteria were histologically or cytologically confirmed non-squamous NSCLC, stage III/IV or recurrent NSCLC not indicated radical chemoradiation, prior one or two regimen of chemotherapy, age 20 years or more, and performance status of two or less. The primary endpoint is objective response rate. The secondary endpoints include overall survival, progression-free survival, disease control rate and incidence of adverse events. This trial plans to accrue 80 patients based on a two-stage design employing a binomial distribution with an alternative hypothesis response rate of 35% and a null hypothesis threshold response rate of 20%. A subset analysis according to EGFR mutation status is planned.

**Discussion:**

We have presented the design of a single arm phase II trial to evaluate the efficacy and safety of combination of bevacizumab and erlotinib in advanced non-squamous NSCLC patients. In particular we are interested in determining the merit of further development of this regimen and whether prospective patient selection using EGFR gene is necessary in future trials.

**Trial registration:**

This trial was registered at the UMIN Clinical Trials Registry as UMIN000004255 (http://www.umin.ac.jp/ctr/index.htm).

## Background

Chemotherapy for advanced non-small-cell lung cancer (NSCLC) patients with good performance status improves survival time and quality of life [1]. Platinum doublet therapies with third-generation agents are thought as the standard in first-line for NSCLC patients, of which response rate is 30-40%, one year survival rate is 26-36% and median survival time is 8-13 months [2-4]. For patients who had relapsed or did not respond to first-line chemotherapy, docetaxel [5-7] and pemetrexed [8] are effective. Erlotinib, an oral epidermal growth factor receptor tyrosine kinase inhibitor (EGFR-TKI), was also shown to improve progression-free survival (PFS) and overall survival (OS) modestly with acceptable toxicity in second- or third-line setting for advanced NSCLC [9,10]. On third-line treatment only erlotinib is recommended by the National Comprehensive Cancer Network guideline [11] and no established treatment options exist for patients who have experienced erlotinib failure.

Several lines of evidence lent support to the notion that combining bevacizumab, a monoclonal antibody targeting the vascular endothelial growth factor (VEGF), with erlotinib for advanced NSCLC might confer additional clinical benefit. Two large phase III trials confirmed that bevacizumab improves survival of advanced non-squamous NSCLC patients when combined with carboplatin plus paclitaxel or cisplatin plus gemcitabine as first-line chemotherapy [12,13]. A significant improvement in PFS and objective response rate (ORR) by the addition of bevacizumab with carboplatin plus paclitaxel was also shown in a randomize phase II trial of Japanese patients [14]. Finally, a recent randomized phase II trial of combination of bevacizumab with erlotinib, combination with cytotoxic drug, and cytotoxic drugalone showed results for PFS and OS favour the combination regimens over cytotoxic drug alone in the second-line setting, although not statistically significant [15].

## Objective

The primary objective of the trial is to evaluate the efficacy and safety of combination of bevacizumab and erlotinib as a second- or third-line chemotherapy for advanced non-squamous NSCLC. Specific hypotheses to be tested are (1) one-sided hypothesis that the ORR of combination of bevacizumab and erlotinib is higher than a pre-specified threshold of 20%, (2) whether this regimen are safe and feasible, and (3) whether the ORR is higher in patients with EGFR mutation than in patients with EGFR wild type.

## Methods

### Design and setting

This study is an open-label, multi-institute, single arm phase II clinical trial. The coordinating office is at Kyoto University Hospital. Registration and data collection are conducted with the use of the web system and the electronic case report form (e-CRF).

### Ethical consideration and registration

The study protocol is according to the Helsinki declaration [16] and the Ethics Guidelines for Clinical Research by the Ministry of Health, Labor, and Welfare [17]. We obtained approval by the ethical committee at Kyoto University on October 27, 2010 (C-453). This trial was registered at the UMIN Clinical Trials Registry as UMIN000004255 (http://www.umin.ac.jp/ctr/index.htm).

### Eligibility criteria

Staging was according to the 7th Edition of the TNM Classification for Lung Cancer [18]. Inclusion criteria are as follows:

1) Histologically or cytologically confirmed non-squamous NSCLC.

2) Stage III/IV or recurrent NSCLC not indicated radical chemoradiation, and prior one or two regimen of chemotherapy.

3) Age 20 years or more at the date of informed consent.

4) The Eastern Cooperative Oncology Group Performance Status of two or less.

5) Presence of measurable lesion.

6) Sufficient hematologic, hepatic, and renal and lung function in laboratory tests 14 days before registration.

7) Expected survival time more than three months.

8) Expected interval more than 28 days after surgery if the patient received a major surgery.

9) Written informed consent by the patient.

Exclusion criteria are as follows:

1) Prior EGFR-TKI.

2) Serious complications.

3) Hemoptysis or bloody sputum of 2.5 mL or more, or history of clinically significant hematemesis, coagulation disorder or thrombosis.

4) A cavitating lesion, a central lesion or a lesion abutting major blood vessels.

5) History of myocardial or cerebral infarction within six months before registration.

6) Refusal of contraception or woman with on-going or contemplating pregnancy or breast-feeding.

7) Brain metastasis with a bleeding risk.

8) Interstitial pneumonia confirmed by computer tomography.

9) Difficulty in ingestion.

10) Pleural effusion which is uncontrolled by local therapy and requires other treatments

11) Patients judged inappropriate for the trial by investigators.

### Patient registration

After confirming eligibility criteria and obtaining informed consent. Eligible patients are registered and then investigators initiate the planned treatment. The accrual started in November 2010 and is to continue for two years.

### Treatment

Patients enrolled in this trial receive the protocol treatment with bevacizumab and erlotinib within 15 days. Dose of the protocol treatment is based on the prior trials [15,19]. Bevacizumab is administered at a dose of 15 mg/kg on the first day of each 3-week cycle. No dose reductions are allowed for bevacizumab. Bevacizumab is terminated if either of the following adverse events occurs.

1) Grade 2 to 4 hemorrhage

2) Grade 3 to 4 thrombosis

3) Delay of administration of each cycle over 23 days

Erlotinib is administered initially at 150 mg/day orally. Tablets are taken at least one hour before or two hours after a meal, preferably in the morning. Dose of erlotinib are reduced by one or two levels of five doses, 150, 125, 100, 75 and 50 mg, if either of the following adverse events occurs.

1) Unacceptable skin toxicity

2) An increase in AST or ALT up to Grade 3 to 4

3) Grade 3-4 diarrhea

Dose escalations for erlotinib are not allowed after a dose reduction. Erlotinib is terminated if either of the following adverse events occurs.

1) Grade 1 to 4 pulmonary fibrosis

2) Grade 4 non-hematologic toxicity other than pulmonary fibrosis

3) Delay from prior administration over 23 days

The protocol treatment is terminated if the disease progresses, serious adverse events occurs or at the patient's refusal. There is no restriction of maximum number of cycles. There is no restriction of treatment after failure of the protocol treatment.

### Endpoints

The primary endpoint is ORR. The secondary endpoints are PFS, OS, disease control rate (DCR) and incidence of adverse events. Patients undergo tumor assessments at baseline and every six weeks by investigators using Response Evaluation Criteria in Solid Tumors version 1.1 [20]. ORR and DCR are defined by the proportion of complete response (CR) and partial response (PR), or the proportion of CR, PR and stable disease (SD), in confirmed best overall response at the time of the primary analysis. OS is defined as the time from registration to death from any cause, and it is censored at the last contact date for living patient. PFS is defined as the time from registration to either the first event of progression of disease or death from any cause, and it is censored at the last date when patient is alive without progression. Adverse events are evaluated according to the Common Terminology Criteria for Adverse Events (CTCAE) version 4.0 [21].

### Data collection

Patients are followed-up for three months after registration. Schedule of data collection are summarised in Table 1. Radiographic data for tumor assessments is collected every six weeks.

**Table 1:** Schedule of data collection

	Baseline	Under treatment	At termination of treatment	After termination of treatment
Physical examination				
Height	○			
Weight, performance status	○	○	○	
Blood pressure	○	○	○	○
Laboratory test				
Blood count	○	○		
Biochemistry test	○	○		
Urine test	○	○		
SpO_2_	○	○		○^*1^
Electrocardiography	○	○^*1^		
EGFR gene	○			
Radiology test				
Chest Xp	○	○^*1^		○^*1^
Chest CT	○	○^*2^		○^*3^
Abdominal CT/Ultra sonography	○	○^*2^		○^*3^
Head CT/MRI	○	○^*1^		○^*1^
Bone scintigraphy/PET	○	○^*1^		○^*1^

*1 If necessary

*2 Every 6 weeks

*3 Six weeks after termination if treatment is terminated for reasons other than progression of disease

### Sample size determination

Simon's minimax two-stage design employing a binomial distribution is used to calculate the required sample size. In the previous trials, the ORR of erlotinib monotherapy ranged 8.9 to 28.3% [9,10]. Thus we consider that an ORR of 20% indicates no value of further investigation of the combination. This trial plans to accrue 42 patients in the first stage and 80 patients in total, which provides 90% power with an alternative hypothesis ORR of 35% and a null hypothesis ORR of 20% using one-sided testing at a 5% significance level.

### Statistical consideration

The analysis population for efficacy is the full analysis set. The primary analysis for efficacy is a one-sided binomial test with the null hypothesis of 20% at a 5% significance level in the second stage. A subset analysis according to EGFR mutation status (direct sequence or PNA-LNA PCR clamp methods) is also planned. ORRs with 95% confidence intervals are calculated in the subsets of mutant and wild type, and compared with 20% using the same binomial test at a 5% significance level separately. Multiplicity is not adjusted for since this is a secondary analysis.

## Discussion

We have presented the design of a single arm phase II trial to evaluate the efficacy and safety of combination of bevacizumab and erlotinib in advanced non-squamous NSCLC patients. In particular we are interested in determining the merit of further development of this regimen and whether prospective patient selection using EGFR gene is necessary in future trials.

## List of abbreviations used

CR: complete response; CTCAE: the Common Terminology Criteria for Adverse Events; NSCLC: non-small-cell lung cancer; e-CRF: electronic case report form; EGFR-TKI: epidermal growth factor receptor tyrosine kinase inhibitor; ORR: objective response rate; OS: overall survival; PR: partial response; PFS: progression-free survival; SD: stable disease; VEGF: vascular endothelial growth factor.

## Competing interests

KY received research funding from Taiho Pharmaceutical and Chugai Pharmaceutical. The other authors declare no competing interests.

## Authors' contributions

KY conceived of the trial. KY, MH, YS, ST and MN designed the trial. MH searched the literature and drafted the protocol. MN supervised the data management and patient registration. ST is responsible for statistical analysis. ST wrote the final manuscript. All authors have read and approved the final manuscript.
